# Migraine aura or transient ischemic attacks? A five-year follow-up case-control study of women with transient central nervous system disorders in pregnancy

**DOI:** 10.1186/1741-7015-5-19

**Published:** 2007-07-17

**Authors:** Janne Marit Ertresvg, Lars Jacob Stovner, Lene Ekern Kvavik, Hans-Jorgen Johnsen, John-Anker Zwart, Grethe Helde, Gunnar Bovim

**Affiliations:** 1 Norwegian National Headache Centre, Department of Neurology and Clinical Neurophysiology, St Olavs Hospital, and Department of Neuroscience, Norwegian University of Science and Technology, N-7006 Trondheim, Norway

## Abstract

**Background:**

Migraine aura may be difficult to differentiate from transient ischemic attacks and other transient neurological disorders in pregnant women. The aims of the present study were to investigate and diagnose all pregnant women with transient neurological disorders of suspected central nervous system origin, and to compare this group with a control group of pregnant women with regard to vascular risk factors and prognosis.

**Methods:**

During a 28 month period, 41 patients were detected with transient neurological symptoms during pregnancy. These were studied in detail with thorough clinical and laboratory investigations in order to make a certain diagnosis and to evaluate whether the episodes might be of a vascular nature. For comparison, the same investigations were performed in 41 pregnant controls. To assess the prognosis, both patients and controls were followed with questionnaires every year for five years.

**Results:**

Migraine with aura was the most common cause of symptoms during pregnancy, occurring in 34 patients, while 2 were diagnosed with stroke, 2 with carpal tunnel syndrome, 1 with partial epilepsy, 1 with multiple sclerosis and 1 with presyncope. Patients had more headache before pregnancy than controls, but the average levels of vascular risk factors were similar. None of the patients or the controls reported cerebrovascular episodes during the five-year follow-up.

**Conclusion:**

The diagnosis of migraine aura was difficult because for many patients it was their first ever attack and headache tended to be absent or of non-migraineous type. The aura features were more complex, with several aura symptoms and a higher prevalence of sensory and dysphasic aura than usual. Gradually developing aura symptoms, or different aura symptoms occurring in succession as described in the International Classification of Headache Disorders, seem to be useful for differentiating aura from other transient disorders. A meticulous history and clinical neurological examination are more useful than routine supplementary investigations for cerebrovascular disease. The five-year follow-up clearly indicates that migraine with aura in pregnancy usually has a good prognosis with regard to cerebrovascular events.

## Background

Pregnancy, as well as migraine, are well-known risk factors for cerebrovascular (CV) episodes (transient ischemic attacks (TIAs) and stroke). The differentiation of TIAs from migraine aura in pregnancy is a rather common clinical problem [1]. Both disorders consist of transient neurological phenomena stemming from the central nervous system (CNS). To complicate the matter, TIAs may be accompanied by headache [2] and migraine aura often occurs without ensuing headache (International Classification of Headache Disorders, second edition (ICHD-2) [3], category 1.2.3 Typical aura without headache). Migraine is particularly common among fertile women, affecting approximately 20–25% of young and middle aged women in Europe [4,5]. Although several studies have shown that migraine improves during pregnancy [6-8], the migraine worsens in some women [9], and some women experience their first migraine attack in this period [10], and particularly migraine with aura (MA) [11-13].

We have previously reported the occurrence of headache and transient neurological symptoms in a large cohort (n = 1,631) of pregnant women based on questionnaires and interviews [14]. The aim of the present study was, with the help of thorough clinical and laboratory investigations and a follow-up, to ascertain the diagnoses for the neurological symptoms, and the CV risk factors in a sample of women with symptoms or signs indicative of transient CNS disorders during pregnancy. We hoped that this would enable us to formulate guidelines on how to differentiate the two disorders among pregnant women.

## Methods

### Subject population

There were 41 patients included in the study during the period from May 1997 to August 1999. The majority, 25 women, were recruited among patients routinely referred to our department from doctors at the Department of Obstetrics and Gynecology, and from general practitioners. The reasons for referral were transient focal neurological deficits where the possibility of migraine aura or CV or other CNS disorders was present. The remainder was recruited from a large prospective cohort study on headache and focal neurological symptoms among pregnant women. This study has been reported previously [14]. In short, 1,631 pregnant women returned screening questionnaires at the time of routine ultrasound screening in week 18 to 20 of the pregnancy about headache and focal neurological symptoms occurring before pregnancy. Right after delivery they were asked to return a similar questionnaire about symptoms occurring during the pregnancy; 278 women reported new symptoms of potential focal neurological character during pregnancy. These were telephone interviewed by a trained doctor in neurology to get more detailed information about the symptoms. Most of these women were deemed to have had symptoms due to disturbances in the peripheral nervous system (for example, carpal tunnel syndrome (CTS)) or symptoms of a non-neurological character. Based on the telephone interview, only 16 (that is, 0.9%. of the total sample) were considered to have had symptoms indicative of CNS disorder and were included in the study for further investigations.

All 41 women (the patients) were subjected to a face to face interview by a trained doctor in neurology and a full clinical and neurological examination. Most were also investigated with electrocardiograms, ultrasound of the precerebral vessels and cerebral magnetic resonance imaging (MRI), as well as extensive blood chemistry screening for coagulation parameters, hemoglobin (Hb), platelet count, erythrocyte sedimentation rate (ESR), activated partial thromboplastin time (APPT), fibrinogen, protein S and C, lupus anticoagulant and anticardiolipin antibodies, homocystein and antithrombin 3. All MRI investigations were performed after delivery. As to the other investigations, in many patients it was not possible for practical reasons to perform them at the same time as the symptoms occurred; thus, in some patients they were done before and in some after delivery. In 16, the blood samples were taken during pregnancy, and in 25 some time after delivery.

To obtain a control group for comparison, one control for each patient (n = 41) was mainly selected from among the 1,631 women in the prospective cohort study without focal neurological symptoms during pregnancy. The controls were included from November 1998 till April 2000. The 41 controls were subjected to the same clinical examination and supplementary investigations as the patient group except for MRI, and they answered the same questionnaires. The controls were matched for age and smoking habits, and with regard to whether they had had deliveries previously or not. For those patients who were still pregnant while we did the clinical examination, the controls were also matched with regard to the stage of the pregnancy. Since the results of several investigations (for example, lab tests, blood pressure) may be influenced by pregnancy, investigations in both the case and the control were performed at the same stage before or after delivery.

All women, both patients and controls, were contacted once a year for five years after inclusion in the study by mail containing a questionnaire asking whether they had contracted any new diseases during the past year, in addition to questions on headache, aura and medication use during the past year. The last follow-up study was carried out in October 2005.

Signed (written) consent was given by all participants. The Regional Committee for Ethics in Medical Research, and the Norwegian Data Inspectorate approved the study.

### Data analysis

Comparisons between patients and controls were performed with Wilcoxon matched pairs signed rank test for continuous variables, and with chi-square test for proportions. *P*-values ≤ 0.05 are considered statistically significant. Data analysis was performed with the Statistical Package for the Social Sciences (SPSS), version 10.0 (Chicago, Illinois).

## Results

### Diagnoses

The final diagnoses for the 41 patients are listed in Table 1. MA was the predominant diagnosis in 34 (83%). In addition, two patients had CTS, two had stroke (one probably related to preeclampsia), one had partial epilepsy, one had multiple sclerosis (MS), and one had presyncope. In the two CTS patients, entrapment of the median nerve was verified by neurography. One of the women with CTS had migraine without aura (MO), but her migraine was not the cause of the transient neurological symptoms. In addition, one MA patient also had clinically probable CTS, but the CTS was not the cause of the neurological symptoms leading to the referral to a neurologist.

**Table 1:** Cause of transient neurological disorder in 41 pregnant women and pathological findings on supplementary investigations

Diagnosis	N (%)	Pathological findings with supplementary investigations
Migraine with aura	34 (82.9)	MRI: one had signs of a previous stroke (incidental finding), two had unspecific white matter lesions ECG: three had right bundle branch block Ultrasound: one had a false positive finding
Stroke	2 (4.8)	MRI: two had changes compatible with recent stroke Blood chemistry: one had positive antiphospholipid antibodies (IgG and IgS), positive antinuclear factor and lupus and lues antibodies; one had elevated fibrininogen, AT3, protein C, and low protein S and APC resistance, and was heterozygous for the factor V Leiden on gene testing
Presyncope	1 (2.4)	Blood chemistry: anemia
MS	1 (2.4)	MRI and cerebrospinal fluid: typical findings for MS
Epilepsy	1 (2.4)	EEG: negative first year, later epileptogenic changes
CTS	2 (4.8)	Median nerve neurography: pathological in both

APC, activated protein C; AT3, antithrombin 3; CTS, carpal tunnel syndrome; ECG, electrocardiography; EEG, electroencephalography; MRI, magnetic resonance imaging; MS, multiple sclerosis.

One of the MA patients probably had a minor stroke ten years before inclusion in the study that had not been investigated at the time. An MRI scan taken after the pregnancy showed signs of an old infarction of the basal ganglia area, deemed not to be related to the migraine aura that occurred during her pregnancy.

The somatic examination was normal in all 41 patients, with the exception of one patient with hypertension due to preeclampsia (vide infra), one with a palpable thyroid gland and one with varices. We found no cases with cervical bruit or heart murmur.

The likelihood of the focal neurological symptoms increased through pregnancy among the MA patients was: In 17%, they occurred during the first trimester, in 32% during the second trimester, and in 51% during the third trimester. The mean week of pregnancy for occurrence of symptoms was similar in those with (n = 34) or without (n = 7) migraine as a cause (26th versus 22nd week, *p *= 0.5, Student's *t*-test).

In the two stroke patients, there was an initial phase with intermittent symptoms that could have been mistaken for migraine, but later the diagnosis was relatively evident. The first woman, 23 years old and previously healthy and without migraine, started to have headache and intermittent episodes of numbness of the right hand after the 30th week of pregnancy. In the 34th week the headache increased in intensity and she developed aphasia and right-sided hemiparesis that subsided gradually over several weeks. Anamnestically, she had several risk factors (heavy smoking, several close relatives with stroke at an early age), and she was heterozygous for the Leiden factor (detected after the stroke). On neurological examination, there were findings corresponding to the symptoms, and computer tomography (CT) and MRI showed signs of left-sided ischemia. Blood chemistry showed increased ESR (56) and low protein S, and MRI angiogram (including venous sequence) and Doppler ultrasound examination showed that pre- and intracerebral vessels were normal.

The second stroke patient was a 33 year old woman in her fourth pregnancy. Her symptoms started in week 19 with epigastric pain, daily headache, neck pain, dizziness and visual obscurations. Two weeks later, she was admitted to hospital with preeclampsia with severe hypertension (240/110 mmHg), proteinuria, and somnolence. A CT scan of the head was normal but the cerebral MRI showed edema and ischemic lesions of both hemispheres. The pregnancy was terminated on maternal indication. The next day she developed paresis of the left hand and was diagnosed with Gerstmann's syndrome with finger agnosia, dyscalculia and left/right confusion. The blood chemistry tests revealed that she had pathological values for homocysteine, antinuclear antibodies (ANA), anticardiolipid antibodies IgG and IgM, lupus anticoagulant and lues antibodies, proving that she suffered from an antiphospholipid antibody syndrome.

The MS patient, a 23 year old woman in her second pregnancy, experienced in the third week of her pregnancy an episode with numbness of the fifth finger on the right hand, lasting 2 hours. Ten days later she developed numbness of both soles of her feet, the right leg and pollakisuria, and a subsequent MRI of the head and spinal fluid examination revealed findings typical of MS.

The patient with epilepsy, a 28 year old woman in her first pregnancy, experienced several episodes lasting from 10 seconds to 1 minute, often starting with numbness in the right arm, cheek and sometimes the foot, and then a feeling of remoteness accompanied by dysphasia (mostly sensory, with paraphasia). Partial complex epileptic seizures were suspected, but electroencephalography (EEG) was normal, as was brain MRI. She was not treated with antiepileptics until four years later when she also developed attacks with tonic arm movements. This time, EEG showed epileptic activity in the right temporal area and she became attack free on lamotrigine.

The patient with probable presyncope, a 28 year old previously healthy woman without migraine or headache complaints, experienced 2 episodes with blurring of vision in the whole visual field, each lasting 10 minutes. Both episodes occurred when she was standing, and both subsided quickly when she lay down. She was anemic (Hb 9.5), had no atherosclerotic risk factors except for the recent delivery, and no other localizing symptoms or signs.

### Migraine diagnoses during pregnancy

With regard to MA subtypes (Table 2), six patients had probable MA (ICHD-2, 1.6.2), the reason for not having definite MA being that they had had only one attack, thus not fulfilling the obligatory criterion of two attacks. Two of these had only aura without headache. One patient had probable basilar-type migraine aura, but the headache phase did not fulfill ICHD-2 criteria of typical MO. Typical aura with non-migrainous headache (ICHD-2, 1.2.2) was the most common migraine subtype, and the lack of accompanying symptoms was the usual reason for not being able to make a diagnosis of typical aura with migraine headache (ICHD-2, 1.2.1). One patient convincingly reported a hemiparesis ipsilateral to sensory symptoms. She had no family history of similar attacks and was classified as sporadic hemiplegic migraine (ICHD-2, 1.2.5).

**Table 2:** Migraine subtype among patients with transient neurological deficit during pregnancy (based on examination and interview by neurologist)

ICHD-2 diagnosis	N (%)
1.2.1 Typical aura with migraine headache	9 (26)
1.2.2 Typical aura with non-migraine headache	12 (35)
1.2.3 Typical aura without headache	5 (15)
1.2.5 Sporadic hemiplegic migraine	1 (3)
1.6.2 Probable MA	6 (18)
1.6.6 Probable basilar-type migraine	1 (3)

### Special features of the migraine aura during pregnancy

The patient with probable basilar-type migraine had several attacks in pregnancy, starting with dizziness followed by bilateral numbness in both hands and feet and the head, and then a feeling like she would faint. After ten minutes she would get a severe throbbing headache without accompanying symptoms. She denied hyperventilation. She had had similar attacks three years before the pregnancy, but without accompanying headache. An EEG at that time was normal.

Among the remaining 33 MA patients, sensory aura was the most common type, occurring in 26 (79%) of them, thereafter followed by visual aura in 20 (61%) and dysphasic aura in 7 (21%). One (2%) also had motor weakness, stating that she could not lift her arm and had paresis of the foot, in addition to a typical sensory aura. Only visual aura occurred in 4 patients (12%), only sensory aura in 12 (36%), and only dysphasic aura in none. A combination of visual and sensory aura occurred in 10 patients (30%), visual and dysphasic aura in 2 (6%), sensory and dysphasic aura in 1 (3%), and visual, sensory and dysphasic aura in 4 (12%). Most patients (31) reported that the symptoms lasted less than 60 minutes per symptom, and 10 patients that the symptoms lasted less than 15 minutes. Two migraine patients could not remember the duration of their aura, but could only say that it had lasted some minutes. In all the others, it had lasted more than five minutes, as required by the ICHD-2 criteria.

The patients with several symptoms all described a gradual progression from one symptom to another, most commonly from visual symptoms to paresthesia. Among the patients with paresthesia, 16 patients reported a march of the symptoms, usually starting in the fingers and spreading to the underarm and ipsilateral lower part of the face, including the mouth. There were 15 patients who experienced positive visual signs, and in 13 of these there was a gradual progression of the symptoms over more than 4 minutes. Of the 20 patients with visual aura, 6 reported that the symptoms started within seconds.

In the MA patient who also had clinically probable CTS, the two disorders were clearly discernible. The migraine episode started with visual signs and continued with numbness of the lower two-thirds of the face combined with numbness of the right arm distal to the elbow. On later examination, there was numbness of the first, second and one-half of the third finger on both hands due to the CTS.

### Migraine and aura before and during pregnancy

A detailed interview on headache and aura features allowing accurate diagnosis before and after the start of pregnancy was performed only in the patient group. Retrospectively, migraine attacks prior to pregnancy were reported by 18 patients, whereof 12 had MA, all of which included visual symptoms. During pregnancy, however, visual symptoms were reported by 20 patients. Only 4 patients reported paresthesia before pregnancy, compared to 26 patients during pregnancy, and speech disturbance was not reported by any patients before pregnancy, but 7 patients experienced this during pregnancy.

### Comparisons of patients and controls

Based on the questionnaires, there was no significant difference between patients and controls with regard to headache before pregnancy (Table 3). Neither were there any significant differences with regard to the presence of the most common risk factors for vascular disorders before or during pregnancy (Table 3), with the exception that the patients had somewhat more previous births than controls.

**Table 3:** Questionnaire data for patients and controls

	Patient group (n = 41)	Control group (n = 41)	*P*-value
Age (years)	28.3	28.9	0.1*
Number of previous births	1.1	0.8	0.02*
Number of previous miscarriages	0.7	0.4	0.1*
Smoking in pregnancy	10	6	0.11^†^
Smoking before pregnancy	18	15	0.16^†^
Headache in pregnancy	38	30	0.04^†^
Headache before pregnancy	36	33	0.5^†^
Oral contraceptives last year before pregnancy	7	7	1.0^†^
Hypertension before pregnancy	0	0	1.0^†^
Hypertension in pregnancy	7	4	0.5^†^
Previous thromboembolic disorder	0	0	1.0^†^
Familial hypertension	18	19	0.9^†^
Angina pectoris	7	7	0.8^†^
Myocardial infarction	14	10	0.4^†^
Stroke	10	7	0.5^†^
Diabetes mellitus	11	13	0.9^†^

*Wilcoxon matched pairs signed rank test; ^†^Chi-square test.

The measured variables are shown in Table 4. Since blood samples were not taken at the same time with respect to the start of pregnancy, and some also with respect to after delivery (range of 5–86 weeks after conception, with a mean of 45 weeks for patients, and 50 weeks for controls), it is not meaningful to compare the values in the different diagnostic groups. However, for each patient and control pair, the blood sample was taken either during pregnancy or after delivery, and at approximately the same time before or after delivery. Comparing the patient and control group (Table 4), patients had significantly higher systolic blood pressure, Hb, and anticardiolipin IgG antibodies and lower glucose. Systolic blood pressure, Hb and glucose were significantly different also when the comparison included only patients who had migraine (n = 34, data not shown), but no significant differences were found among patient-control pairs when the patient did not suffer from migraine. Comparing the proportions of patients and controls with pathological findings, or the proportions among MA patients and their respective controls, there were no statistically significant findings (Table 4).

**Table 4:** Various measurements in patients and controls as means (SD) and number of pathological values

			Number with pathological values	
			
	Patients	Controls	Patient group	Controls/Mi controls
Pulse	78 (14)	72 (12)*	2 >100 beats/min (1 CTS, 1 Mi)	0/0
Systolic blood pressure	120 (15)	112 (10)^†^	1 >150 mmHg (stroke)	0/0
Diastolic blood pressure	74 (10)	72 (8)	1 >95 mmHg (Mi)	0/0
Hemoglobin	12.9 (1.1)	12.3 (1.1)^†^	4 low (all Mi)	10/9 low
Platelet count	250 (74)	249 (63)	1 low (MS), 2 high (stroke, Mi)	2 low, 1 high/2 low, 1 high
ESR	20 (19)	16 (12)	13 high (2 CTS, 1 stroke, 1 PS, 9 Mi)	9/8 high
APPT	27.7 (5.0)	27.6 (2.2)	2 high (stroke, Mi)	0/0
Fibrinogen	3.8 (1.3)	3.5 (0.9)	12 high (1 stroke, 1 PS, 2 CTS, 8 Mi)	8/7 high
Antithrombin 3	112 (15)	108 (15)	1 low (Mi)	3/3 low
Cholesterol	5.4 (1.2)	5.2 (1.3)	5 high (1 CTS, 4 Mi)	3/3 high
Triglycerides	1.5 (1.1)	1.1 (0.8)	6 high (1 CTS, 5 Mi)	3/3 high
Glucose	4.6 (0.5)	5.0 (0.9)*	0	3/2 high
Anticardiolipin ab IgG	0.16 (0.4)	0.03 (0.2)	7 positive (1 stroke, 6 Mi)	1/1 positive
Anticardiolipin ab IgM	0.14 (0.4)	0.05 (0.2)	4 positive (1 stroke, 3 Mi)	3/2 positive
Antinuclear antigen	0.11 (0.3)	0.11 (0.3)	5 positive (1 stroke, 4 Mi)	4/3 positive
Lupus anticoagulant	0.3 (0.2)	0.0 (0)	1 positive (stroke)	1/1 positive
Lues serology	0.3 (0.2)	0.0 (0)	1 positive (stroke)	0/0
Homocysteine	9.9 (6.0)	9.3 (7.3)	6 high (1 stroke, 5 Mi)	1/0 high
Protein C	113 (20)	108 (15)	0	0/0
Protein S	71 (23)	68 (15)	16 low (1 stroke, 2 CTS, 13 Mi)	18/15 low

*P ≤ 0.05; ^†^*p *≤ 0.01 (Wilcoxon matched pairs signed rank test). There were no significant differences when comparing the number of pathological values between patients and matched controls (n = 41 in each group, chi-square test) or migraineurs and their matched controls (n = 34 in each group). Ab, antibodies; APPT, activated partial thromboplastin time; CTS, carpal tunnel syndrome; ESR, erythrocyte sedimentation rate; Mi, migraine; MS, multiple sclerosis; PS, presyncope.

In the five-year follow-up study, there were some missing data. Of the patient group, 1 patient did not answer any contact, and 3 only answered in the first year, and 35 answered at least once after 4 or 5 years. Of the control group, all women answered at least once, 1 answered only the first year, and 39 answered at least once after 4 or 5 years. Of the patients, four developed asthma, two hypertension, one hypothyreodism, and one arthritis. All disorders occurred among the migraine patients. Of the controls, one woman got asthma and one psoriasis. None reported CV episodes in either group. The numbers reporting a new disease were not statistically different (chi-square test, *p *= 0.09).

## Discussion

The present study clearly shows that MA, definite or possible, was the dominant cause of transient focal symptoms or signs during pregnancy, occurring in 34 of the 41 patients (76%). Extensive investigations among the migraineurs to detect possible CV disease were negative, and none of them reported CV disorders during the five-year follow-up. Other causes of transient focal symptoms were CTS, occurring in two patients as the main cause and additionally in one in whom migraine aura was the main cause for referral. Furthermore, there were cases of partial epilepsy, multiple sclerosis, stroke and (probable) presyncope.

When a patient presents with transient CNS symptoms during pregnancy, a main concern for the doctor is whether the episode represents a transient ischemic attack, heralding a stroke. Therefore, we evaluated routinely a series of risk factors for CV disease under the assumption that if a high proportion of the episodes were of such a nature, the patient group would, in general, have higher risk factor scores than the control group. In retrospect, it appeared that only two patients had CV episodes. This low prevalence was corroborated by the fact that patients and controls were rather similar with regard to risk factors, both self-reported (Table 3) and measured (Table 4). Although investigations were carried out at the same stage for each patient and control, some of the significant differences between patients and controls may be explained by the fact that, for some of the patients, the blood samples or measurements were taken when they were in the hospital in the acute phase, and they were thus possibly more stressed (increased pulse and systolic blood pressure), dehydrated (higher haemoglobin) or had fasted for a longer time (low glucose). It has also been shown that high Hb may be a risk factor for migraine and headache [15], which also may explain the higher Hb levels among patients, the large majority of whom were migraineurs.

The value of the supplementary diagnostic investigations in each individual was relatively limited. However, routine blood samples were useful in the two stroke patients. In the stroke patient with an antiphospholipid syndrome, the diagnosis was suspected on the basis of clinical and increased anticardiolipin IgG and lupus anticoagulant. The other stroke patient had low protein S and activated protein C resistance, indicating the presence of factor V Leiden, which was confirmed by genetic testing (the patient was heterozygous for it). A fall in free protein S is a physiological adaption in a normal pregnancy [16].

Routine MRI was useful for diagnosing stroke and MS, but in these patients, the clinical course indicated a serious CNS disorder at a relatively early stage. MRI could also definitively diagnose a previous minor stroke that had no relation to the migraine aura in pregnancy. Neurography was useful for confirming the diagnosis of CTS, but the clinical distinction from migraine aura was quite easy to make even in the case with MO and comorbid CTS. Routine use of Doppler investigation of intra- and precerebral arteries was not helpful diagnostically in the present series.

There were probably several reasons why the episodes of MA seen in this series caused concern about more serious CNS disorders. Firstly, in many patients it was the first and only attack, allowing only a probable MA diagnosis to be made. Secondly, many had typical aura without headache or with non-migraineous headache, making the migraine diagnosis less obvious. Thirdly, the aura symptoms tended to be somewhat atypical, with more patients having sensory than visual aura, many having speech problems, and many having several aura symptoms in succession. This is in contrast to a previous study in a non-pregnant unselected population where 70% of MA patients had visual aura without other symptoms [17]. One can not, however, conclude from the present study that the aura in pregnant women is different from that of the general migraine population since most of the patients in our series were from a highly selected group referred to a neurologist due to suspicion of a more serious disorder.

It has previously been convincingly demonstrated that migraine, and particularly MA, is an independent risk factor for CV disease, both in young and middle-aged women [18-21]. The fact that none of the migraine patients suffered CV episodes during the remainder of the pregnancy, in the post partum phase or during the five years of follow-up may serve as additional evidence that the migraine diagnosis was correct in these patients, and it also clearly indicates that MA in pregnancy is usually a benign condition, both in the short and the long term. However, due to the relatively low number of migraine patients in the present study and the rarity of stroke in this age group, the present study cannot be taken as evidence against the previous studies showing that MA is a relatively weak risk factor for stroke.

Several previous smaller studies have indicated that although migraine in general tends to improve in pregnancy, MA is more common and may quite often occur for the first time during this period. In one study [13], 50–70% of women with migraine improved during pregnancy, but a small proportion (4–8%) experienced worsening of their migraine, particularly those with MA. In addition, migraine started *de novo *during pregnancy in approximately 10% of the cases [22], and the new-onset migraine was often MA. In another study [23], seven out of eight pregnant women with focal neurological symptoms had headache compatible with migraine, and three for the first time during pregnancy. It was concluded that migraine may certainly present in pregnancy for the first time, particularly MA. Another small study reported that MA was more common than expected in patients having migraine occurring for the first time in pregnancy (eight of the nine patients) [11]. Five of these MA patients had visual disturbances, five had paresthesia and four had dysphasia; these proportions are similar to those in the present study.

From the present study it is not easy to abstract guidelines on how to differentiate migraine aura from TIAs in pregnancy since there were only two patients with vascular episodes. In the patient with preeclampsia and malignant hypertension, the transient visual obscurations that occurred some weeks before the stroke should not be classified as a TIA due to brain ischemia. More likely, it was due to brain edema caused by increased intracranial pressure. The transient episodes in the stroke patients were not characterized by a gradual progression of the symptom, as was the case with most of the migraine auras. In addition, all the migraine patients (except two who could not remember) had aura lasting more than five minutes. This may also be useful for differentiating the condition from TIA, which is often even shorter. Hence, the features that are highlighted in the ICHD-2 criteria for migraine aura – a gradual increase in visual and sensory symptoms or a succession of symptoms over more than five minutes – seems to be characteristic, at least in the present case series. Gradual development is not required for the dysphasic aura, but in the present series of patients, this never occurred alone as an aura symptom.

## Conclusion

The main conclusions of the present study are that MA is by far the most common disorder underlying transient CNS symptoms among pregnant women referred to a neurologist in our setting. Sensory and dysphasic aura, and aura with several symptoms, or without headache, seem to be more common in this context than among MA patients in general. More rarely, similar symptoms may herald other disorders, like stroke, MS or epilepsy. The study also documents that the prognosis of MA in pregnancy with regard to later CV events tends to be good. Knowledge of typical migraine aura features combined with a thorough history and clinical evaluation is usually sufficient to differentiate migraine aura from other disorders, but blood chemistry for vascular risk factors and brain imaging studies are useful in cases where there is still doubt about the diagnosis.

## Competing interests

The author(s) declare that they have no competing interests.

## Authors' contributions

Janne Marit Ertresvåg participated in the conception and design of the study, the acquisition of data, the interpretation and analysis, and in drafting and revising the manuscript. Lars Jacob Stovner participated in the interpretation and analysis, and in drafting and revising the manuscript. Lene Ekern participated in the acquisition of data, the interpretation and analysis, and in drafting the manuscript. Hans-Jørgen Johnsen participated in the acquisition of data, and in drafting and revising the manuscript. John-Anker Zwart participated in the acquisition of data, and in drafting and revising the manuscript. Grethe Helde participated in the conception and design of the study, acquisition of data, and in drafting and revising the manuscript. Gunnar Bovim participated in the conception and design of the study, and in drafting and revising the manuscript.

## Pre-publication history

The pre-publication history for this paper can be accessed here:


